# New Insights into Hybrid Materials Based on Conductive Polymers and Their Use in Energy-Related Applications

**DOI:** 10.3390/ma15144928

**Published:** 2022-07-15

**Authors:** Marie-Pierre Santoni

**Affiliations:** Université Paris Cité, CNRS, ITODYS, F-75013 Paris, France; marie-pierre.santoni@univ-paris-diderot.fr

This Special Issue in *Materials* aims to gather both articles and reviews that report the recent progress in the development of electronic hybrid materials based on conductive polymers, with designed structures and tunable properties for applications ranging from energy harvesting (piezoelectrics, thermoelectrics, etc.), to conversion (photovoltaics, (photo) electrocatalysis, etc.) and storage (supercapacitors, batteries, etc.).

Over the past decades, conductive polymers have attracted much interest from scientists in various applicative fields, due to their tunable structures/properties and their easy processability [1,2,3,4]. In particular, the class of electronically conductive polymers, discovered in the 1970s by Alan J. Heeger, Alan G. MacDiarmid and Hideki Shirakawa (Nobel Prize in Chemistry, 2000), continues to be the focus of intense research for novel materials. Indeed, the tunable mechanisms of charge transfer and charge transport processes occurring at the redox sites of conducting polymeric materials, can be advantageously used in a wide range of applications for optoelectronic devices, ref. [5] energy storage, electrocatalysis, photoelectrochemistry, organic- and bio-electrochemistry, electroanalysis, sensors, electrochromic displays, multi-functional coatings, etc.

In light of global sustainable development and solar energy conversion/storage, advanced hybrid materials engineering is one of the key areas to develop clean, low-cost and stable energy technologies. Several pathways are explored and contributions from this Special Issue highlight some of them, using conducting polymers in different areas. Importantly, challenges that are common to various approaches have emerged for the development of devices. In particular, the conversion efficiency of the light-to-charge transducing technologies remains hampered by detrimental losses (recombination of charges) and degradation processes within the materials and the devices, despite their promising potential and important advances in the past decades.

In order to overcome these drawbacks, a widely used strategy relies on carefully chosen components and their individual optimization, each targeting a specific function within the device (for example, light absorption, exciton splitting, charge carrier extraction). By combining the advantages of components with careful integration in the material, it is possible to significantly improve the performance of the device. Understanding the correlation of device performance with material properties, further developing their processing technology and mastering their integration into functional devices represent a timely, challenging and dynamic multidisciplinary field of research. As a recent example, a strategy developed by Liu et al. for OPV (Organic PhotoVoltaics) relies on a new material made of a quaternary blend mixture [6]. The electronic structure and morphology of each of the four components of the mixture were fine-tuned using molecular engineering techniques, with each of them working in synergy to avoid performance trade-offs on the three primary metrics of power conversion efficiency. This strategy ultimately improved the performances of the devices, reaching a power conversion efficiency of 18.07%.

In the last few centuries, we probably consumed a large amount of the energy stored by Nature as fossil fuels over a period of millions of years. Just as important as this (if not more), we may have drawn a large portion of groundwater which is a precious resource that was stored over thousands of years. This has triggered some global and noticeable changes, which were once thought to be “without effect on us”. What will be left for the next generations? What will we do now, for our present and our future? It is up to us all to provide answers to these questions together, and the sooner the better.
